# Circular RNA circUBXN7 represses cell growth and invasion by sponging miR-1247-3p to enhance B4GALT3 expression in bladder cancer

**DOI:** 10.18632/aging.101573

**Published:** 2018-10-12

**Authors:** Hongwei Liu, Dongliang Chen, Junming Bi, Jinli Han, Meihua Yang, Wei Dong, Tianxin Lin, Jian Huang

**Affiliations:** 1 Department of Urology, Sun Yat-sen Memorial Hospital, Sun Yat-sen University, Guangzhou 510120, China; 2 Guangdong Provincial Key Laboratory of Malignant Tumor Epigenetics and Gene Regulation, Sun Yat-sen Memorial Hospital, Sun Yat-sen University, Guangzhou 510120, China; 3 Department of Medical Oncology, Sun Yat-sen University Cancer Center, Guangzhou 510060, China; 4 State Key Laboratory of Oncology in South China, Collaborative Innovation Center for Cancer Medicine, Guangzhou 510060, China; * Equal contribution

**Keywords:** circular RNA, circUBXN7, circ0001380, miR-1247-3p, B4GALT3, bladder cancer

## Abstract

Circular RNAs (circRNAs) have recently been confirmed to participate in different pathological processes, including cancer progression. However, the role and precise mechanism of action of the majority of circRNAs have not been elucidated in bladder cancer (BC). Here, we identified a novel circular RNA, termed circUBXN7, which was significantly downregulated in BC tissues compared with matched nontumor tissues. Importantly, we found that decreased circUBXN7 expression was associated with pathological stage, grade and poor prognosis of BC patients. Functional experiments showed that circUBXN7 overexpression dramatically inhibited proliferation, migration and invasion *in vitro* and suppressed tumor growth *in vivo*. Mechanistically, circUBXN7 could directly bind to miR-1247-3p and reverse the oncogenic effects induced by miR-1247-3p. Furthermore, B4GALT3 was predicted and confirmed to be a target of miR-1247-3p. Rescue experiments demonstrated that circUBXN7 abrogated miR-1247-3p-mediated inhibition of B4GALT3 expression. Finally, silencing of B4GALT3 promoted proliferation and invasion of BC cells; and partially abolished the tumor suppressive effects caused by circUBXN7. Taken together, our study revealed that circUBXN7 serves as a competitive endogenous RNA of miR-1247-3p to elevate B4GALT3 expression, consequently inhibiting cell viability and invasion in BC. The circUBXN7-miR-1247-3p-B4GALT3 regulatory network may provide a new perspective for gene-based treatment strategies for BC.

## Introduction

Bladder cancer (BC), one of the most common malignancies worldwide, affected approximately 3.4 million people globally from 1990 to 2015, with 430,000 new cases diagnosed every year [1], and poses a great threat to human health. Though substantial advances in the combined strategies of surgery and adjuvant therapy have been made in recent years, the recurrence rates for nonmuscle invasive bladder cancer (NMIBC) remain obstinately high [2], and the 5-year overall survival rate remains low [3]. One of the most important factors resulting in a high recurrence rate and poor prognosis is an inadequate understanding of the biological and molecular mechanisms of BC development and progression. Therefore, to improve the prognosis of BC patients, there is an imperative need to identify novel molecular targets and to explore critical molecular mechanisms of BC.

To date, different types of aberrantly expressed molecules including immune-related genes [4], microRNAs (miRNAs) [5] and long noncoding RNAs (lncRNAs) [6] have been reported to take part in the regulation of BC progression. However, molecules involved in tumorigenesis and progression remain largely undiscovered and poorly elucidated. In the last few years, circular RNAs (circRNAs) have attracted considerable attention for their involvement in the development and progression of cancer due to their characteristics of relative abundance, high stability and evolutionary conservation among different tissues [7,8]. CircRNAs represent a novel class of endogenous noncoding RNAs (ncRNAs) that are characterized by covalently-closed loop structures without a 5’-cap or 3’-polyadenylated tail [9]. Mounting evidence has revealed that circRNAs are involved in a variety of biological processes, such as cell proliferation [10], metastasis [11] and angiogenesis [12]. However, although a few circRNAs such as circHIPK3 [13], circ-ITCH [14], and circular RNA MYLK [15], have been reported to play vital roles in BC, until now, the function and mechanism of action of the majority of BC-related circRNAs have not been clearly elucidated.

CircUBXN7 (hsa_circ_0001380), one of the most dysregulated circRNAs identified through high-throughput sequencing in BC and normal tissues [16], has not yet been reported in any types of human cancers. In the present study, we discovered that circUBXN7 was significantly downregulated in BC tissues and was associated with the pathological T stage, grade and prognosis. Functionally, circUBXN7 inhibited BC cell proliferation, migration and invasion *in vitro* and suppressed tumor growth *in vivo*. Mechanistically, circUBXN7 serves as a tumor suppressor by sponging miR-1247-3p to upregulate B4GALT3 in BC. Our findings revealed that circUBXN7 may act as a promising therapeutic target for BC.

## RESULTS

### Characterization of circUBXN7 in BC cells

**Figure 1:**
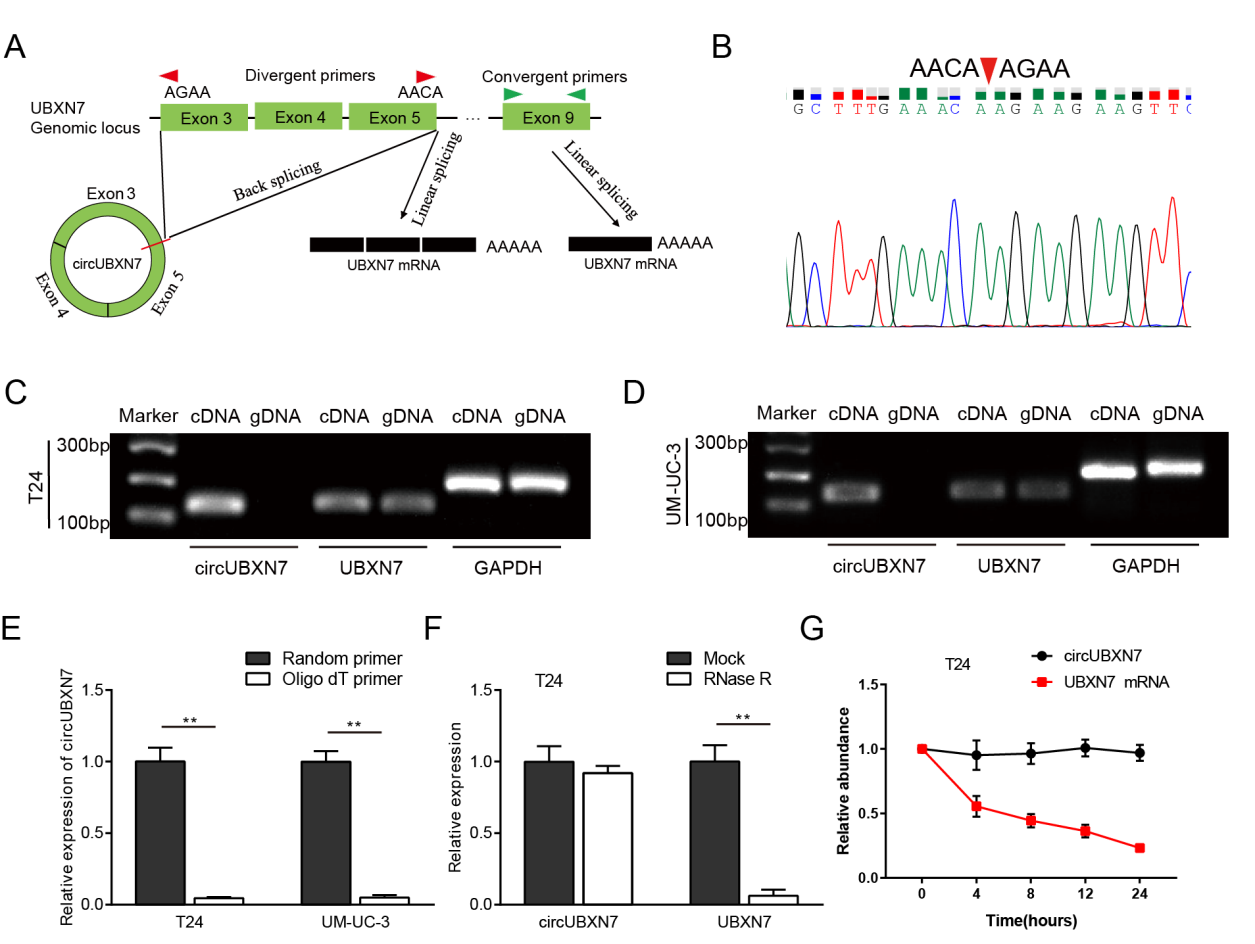
**The generation and characterization of circUBXN7.** (**A**) The diagram shows that circUBXN7 is back-spliced from exon 3 and exon 5 of precursor UBXN7 mRNA, and linear UBXN7 is generated by canonical linear splicing. (**B**) The product amplified by divergent primers was confirmed by Sanger sequencing. (**C and D**) The products amplified using convergent or divergent primers were verified by agarose gel electrophoresis. GAPDH was used as a linear control. (**E**) CircUBXN7 levels were detected by qRT-PCR using random or oligo (dT) primers for reverse transcription. (**F**) qRT-PCR analysis was performed to test circUBXN7 expression in T24 cells with or without RNase R treatment. (**G**) Total RNA harvested from T24 cells with Actinomycin D treatment at the indicated time points was subjected to qRT-PCR. **P<0.01.

According to the circBase database and UCSC, circUBXN7 is generated from exons 3, 4 and 5 of UBXN7 (chr3:196118683-196129890), with exon 3 and exon 5 back-spliced (247 bp). Next, to characterize circUBXN7, we designed two kinds of primers: the divergent primers spanning the back-splice junction site of circUBXN7 were applied to detect the circUBXN7, and the convergent primers from exon 9 of UBXN7 were used to amplify the linear transcripts (Figure 1A). Both complementary DNA (cDNA) and genomic DNA (gDNA) were used as templates during the amplification process. PCR and subsequent agarose gel electrophoresis showed that after divergent primers were used, distinct product of the expected size was amplified from cDNA but not from gDNA and was further confirmed by Sanger sequencing (Figure 1B-1D). A linear UBXN7 transcript was amplified from both cDNA and gDNA using convergent primers (Figure 1C and 1D). Circular RNAs have covalently-closed continuous loop structures with neither 5’ or 3’ polarity nor a polyadenylated tail [9]. To further confirm their circular characteristics, random or oligo (dT) primers were used in the reverse transcription experiments using extracted RNA from T24 and UM-UC-3 cells. The qRT-PCR results showed that circUBXN7 expression was significantly lower when oligo (dT) primers were used, than when random primers were used (Figure 1E), indicating that circUBXN7 had no poly-A tail. Moreover, in contrast to linear UBXN7 mRNA, circUBXN7 was resistant to RNase R, a processive 3’ to 5’ exoribonuclease that degrades linear RNAs but does not digest circRNAs (Figure 1F). Furthermore, actinomycin D, an inhibitor of transcription, was applied to treat T24 cells, and then RNA was harvested at the indicated time for detection of circUBXN7 levels. The results showed that circUBXN7 exhibited a half-life more than 24 hours, whereas the half-life of the linear UBXN7 mRNA was less than 8 hours (Figure 1G). Collectively, these data confirmed that circUBXN7 is an exonic circRNA that is an abundant, circular and highly stable transcript of UBXN7 in BC cells.

### Downregulated circUBXN7 predicts a poor prognosis of BC patients

**Figure 2:**
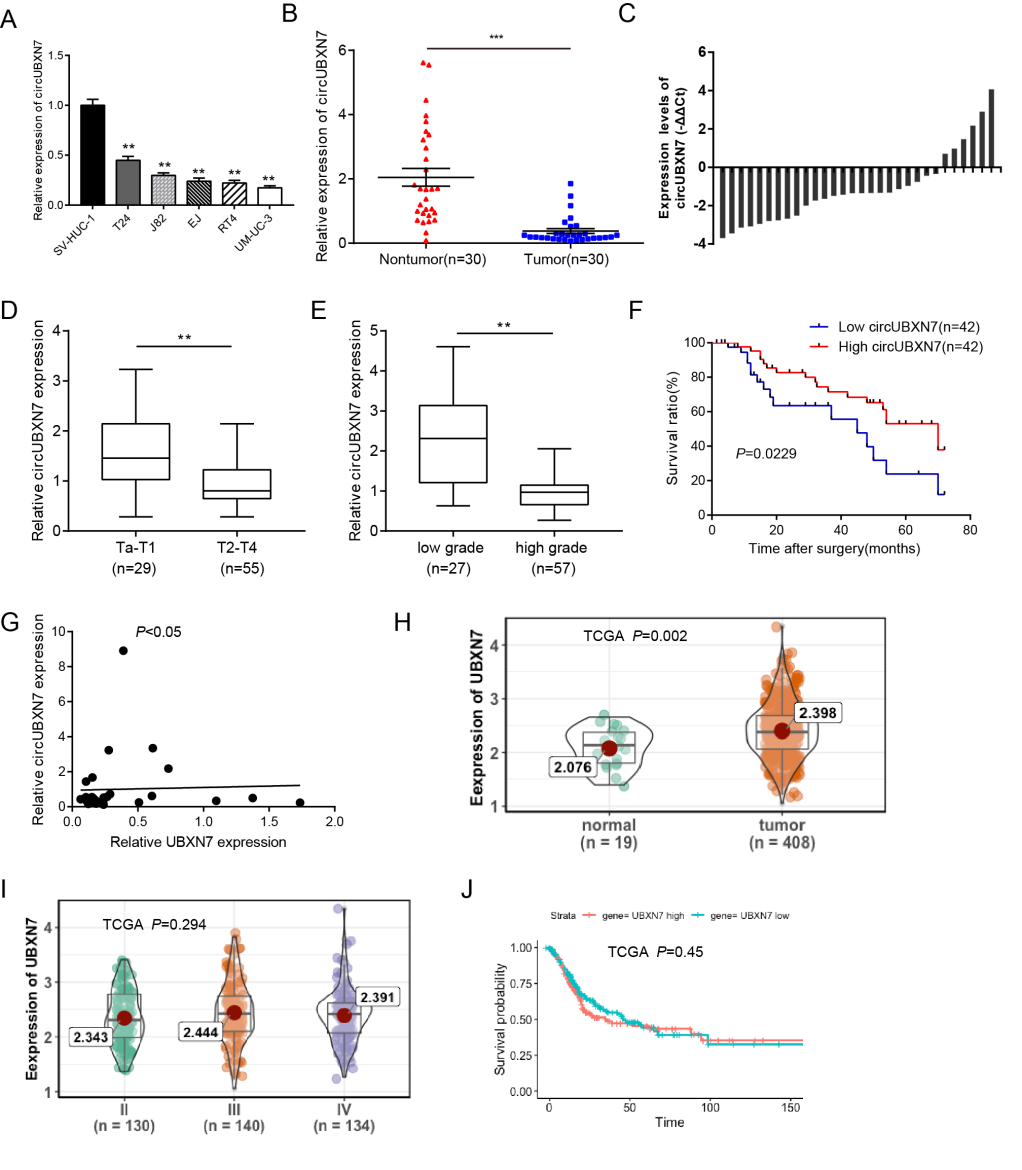
**CircUBXN7 was downregulated in BC tissues and cells.** (**A**) The expression of circUBXN7 was determined by qRT-PCR in BC cell lines. SV-HUC-1 was used as a normal control. (**B**) The expression of circUBXN7 normalized by GAPDH was detected by qRT-PCR in BC tissues (n=30) and adjacent nontumor tissues (n=30). (**C**) qRT-PCR analysis of circUBXN7 levels calculated by -ΔΔCt (ΔCt (cancer)-ΔCt (nontumor)). (**D**) qRT-PCR results demonstrated that circUBXN7 expression was lower in T_2_-T_4_ BC tissues than in T_a_-T_1_ tissues. (**E**) CircUBXN7 expression was lower in high-grade tissues than that in low-grade tissues. (**F**) The Kaplan-Meier method was applied to evaluate the overall survival rate in BC patients. (**G**) Correlation analysis of circUBXN7 levels with UBXN7 mRNA levels in 30 BC tissues. (**H**) TCGA database analysis of UBXN7 mRNA levels in human BC normal tissues (n=19) and tumor tissues (n=408). (**I**) TCGA analysis of UBXN7 mRNA levels in different stages of BC. (**J**) The Kaplan-Meier survival analysis of low and high level of UBXN7 from TCGA database. *P<0.05, **P<0.01, ***P<0.001.

To explore the role of circUBXN7 in BC, we first validated its expression levels in BC cell lines (T24, J82, EJ, RT4, and UM-UC-3). As shown in Figure 2A, the relative expression levels of circUBXN7 were dramatically decreased in BC cell lines compared to a normal urothelial cell line SV-HUC-1. Then, we examined the expression of circUBXN7 in 30 pairs of BC tissues and adjacent nontumor tissues using qRT-PCR. Consistently, circUBXN7 expression was significantly downregulated in 22 out of 30 (73.33%) BC samples, with a median difference of approximately 8.67-fold (Figure 2B and 2C). To further investigate whether the circUBXN7 expression levels correlate with clinicopathological characteristics in BC, another 54 cases of BC tissues were used in this study. By analyzing circUBXN7 levels in a total of 84 BC tissues, we found that the expression of circUBXN7 was significantly decreased in BC tissues with T_2_-T_4_ stages compared to tissues with T_a_-T_1_ stages (Figure 2D). Moreover, the high-grade BC tissues had lower circUBXN7 levels than low-grade ones (Figure 2E). Subsequently, a total of 84 patients was classified into high circUBXN7 expression group (n=42) and low circUBXN7 expression group (n=42) according to the median expression value. The Chi-square analysis demonstrated that decreased circUBXN7 expression was correlated with a high pathological T stage and a high grade. However, no other parameters, such as age, gender, tumor size, lymphatic metastasis or vascular invasion, were significantly different between the two groups (Table 1). Furthermore, the Kaplan-Meier analysis and log-rank test revealed that patients with low levels of circUBXN7 had a shorter overall survival rate than those with high circUBXN7 expression (Figure 2F).

**Table 1:** Correlation between circUBXN7 expression and clinicopathological characteristics in bladder cancer.

		**circUBXN7 expression**			
**Characteristics**	**No. (%)**	**Low(%)**	**High(%)**		***P*-value**
Gender					
Male	51(60.7)	23(45.1)	28(54.9)		0.264
Female	33(39.3)	19(57.6)	14(42.4)		
Age					
<60	31(36.9)	18(58.1)	13(41.9)		0.258
≥60	53(63.1)	24(45.3)	29(54.7)		
Tumor size					
<3cm	48(57.1)	20(41.7)	28(58.3)		0.078
≥3cm	36(42.9)	22(61.1)	14(38.9)		
Pathology stage					
pT_a_-pT_1_	29(34.5)	9(31.0)	20(69.0)		0.012*
pT_2_-T_4_	55(65.5)	33(60.0)	22(40.0)		
Grade					
Low	27(32.1)	7(25.9)	20(74.1)		0.002*
High	57(67.9)	35(61.4)	22(38.6)		
Lymphatic metastasis					
Yes	46(54.8)	27(58.7)	19(41.3)		0.079
No	38(45.2)	15(39.5)	23(60.5)		
Vascular invasion					
Yes	11(13.1)	8(72.7)	3(27.3)		0.106
No	73(86.9)	34(46.6)	39(53.4)		
Total	84	42	42		

Chi-square test. * *P*<0.05

Both circUBXN7 and UBXN7 mRNA are derived from the same precursor RNA of UBXN7 gene through back splicing and canonical linear splicing respectively (Figure 1A), prompting us to investigate whether the circUBXN7 levels in BC tissues were correlated with the corresponding levels of UBXN7 mRNA. The correlation analysis showed that UBXN7 mRNA levels were poorly correlated with circUBXN7 levels in 30 BC tissues (Figure 2G). Further analyses from TCGA demonstrated that UBXN7 levels were significantly higher in 408 BC tissues compared with 19 normal tissues (Figure 2H). However, UBXN7 mRNA levels showed no statistically significant at different BC stages (Figure 2I). In addition, there was no significant difference in the overall survival rate between the high UBXN7 level group and the low UBXN7 level group (Figure 2J). Therefore, we only focused on circUBXN7 for further study.

### CircUBXN7 inhibits proliferation, migration and invasion of BC cells *in vitro*

**Figure 3:**
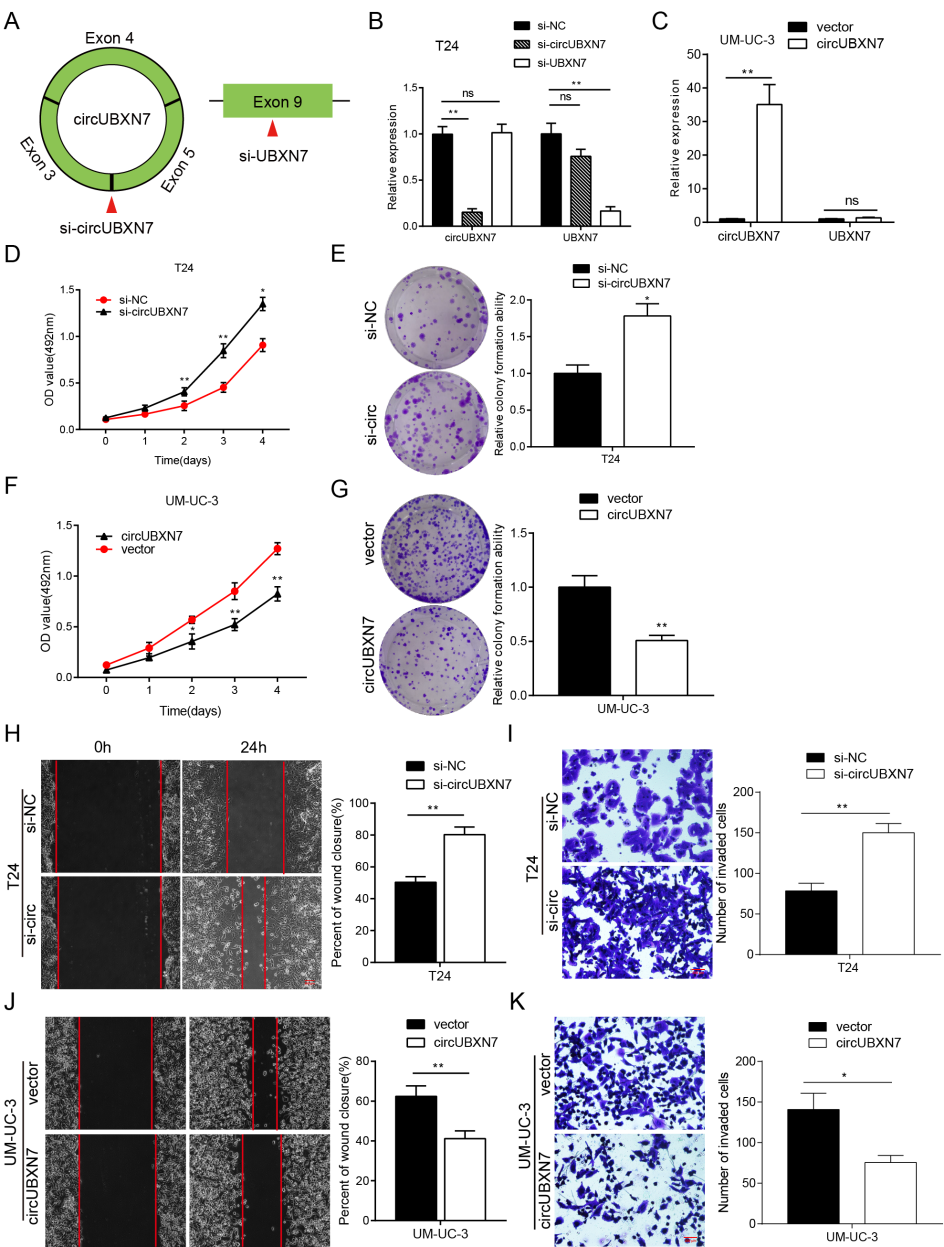
**CircUBXN7 exerted a tumor suppressive role in BC cells.** (**A**) Schematic of the silencing sites of circUBXN7 and linear UBXN7. (**B**) Relative expression of circUBXN7 and UBXN7 mRNA after knockdown of circUBXN7 or UBXN7 examined by qRT-PCR. (**C**) Relative expression of circUBXN7 and UBXN7 mRNA after overexpression of circUBXN7. (**D**) MTS cell viability assay of T24 cells treated with si-circUBXN7. (**E**) Cell clone numbers were counted when circUBXN7 was silenced in T24. (**F**) Overexpression of circUBXN7 inhibited cell growth in UM-UC-3 cells detected by MTS assay. (**G**) CircUBXN7 overexpression reduced clone numbers of UM-UC-3 cells tested by colony formation assay. (**H**) Silencing of circUBXN7 enhanced cell migration of T24 cells performed by wound healing assay. Scale bar, 500 μm. (**I**) Knockdown of circUBXN7 increased the invasive ability of T24 cells as detected by transwell Matrigel invasion assay. Scale bar, 200 μm. (**J**) CircUBXN7 overexpression impaired migratory capacity of UM-UC-3 cells. (**K**) CircUBXN7 overexpression repressed invasive ability in UM-UC-3 cells. *P<0.05, **P<0.01.

To gain insights into the biological function of circUBXN7, we detected the expression of circUBXN7 in diverse human BC cell lines using qRT-PCR. The highest level of endogenous circUBXN7 was verified in T24; a moderate level was detected in J82, EJ and RT4; and the lowest expression was found in UM-UC-3 cell lines (Figure 2A). Thus, T24 and UM-UC-3 cell lines were chosen for the loss-of-function experiments and gain-of-function assays, respectively. We first designed one siRNA to specifically target the back-splice junction of circUBXN7; and another siRNA that only targets exon 9 of linear UBXN7 (Figure 3A). After T24 cells were transfected with si-circUBXN7, the expression of circUBXN7 was successfully downregulated, whereas the linear UBXN7 mRNA level was slightly decreased, but no statistical significance was observed. Additionally, si-UBXN7 remarkably knocked down linear UBXN7 expression without influencing circUBXN7 level (Figure 3B). Moreover, an ectopic overexpression vector of circUBXN7 was successfully constructed, and after overexpressing circUBXN7 in UM-UC-3, the level of circUBXN7 was dramatically increased without significant increase in linear UBXN7 level (Figure 3C).

Then, we conducted MTS and colony formation assays to delineate the effects of circUBXN7 on BC cell viability and proliferation. The results of the MTS assays revealed that the specific circUBXN7 knockdown significantly promoted viability of T24 cells (Figure 3D). Similarly, colony formation assays demonstrated that downregulation of circUBXN7 strikingly increased the colony-forming ability of T24 cells (Figure 3E), while overexpression of circUBXN7 markedly repressed the viability and proliferation of UM-UC-3 cells (Figure 3F and 3G). Furthermore, the wound healing and transwell Matrigel assays demonstrated that circUBXN7 knockdown apparently enhanced migratory and invasive potential in T24 cells (Figure 3H and 3I). In contrast, overexpression of circUBXN7 significantly impaired the migratory and invasive capacities of UM-UC-3 cells (Figure 3J and 3K). Taken together, circUBXN7 functions as a tumor suppressor in BC.

### CircUBXN7 directly binds to miR-1247-3p and suppresses miR-1247-3p activity

**Figure 4:**
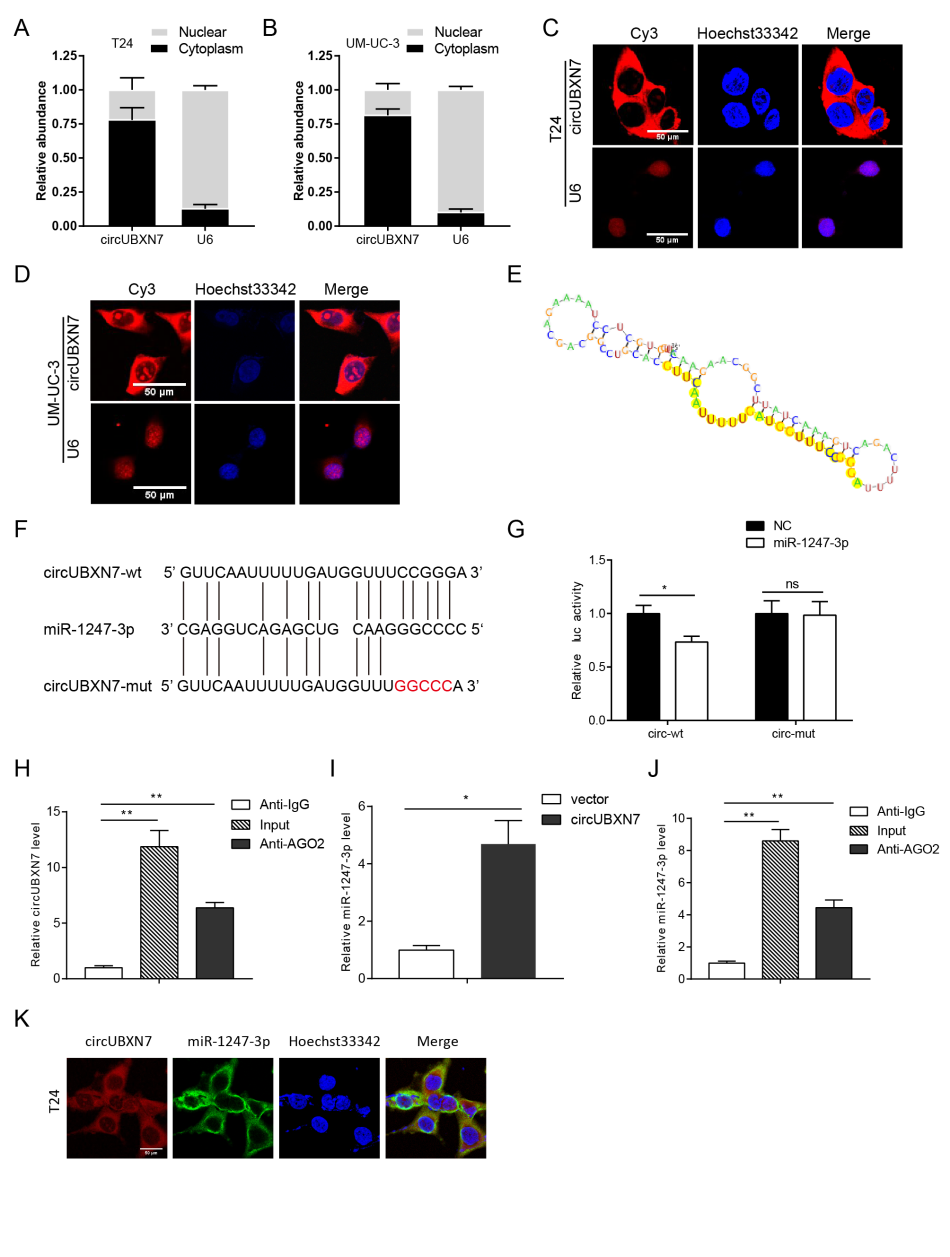
**CircUBXN7 directly bound to miR-1247-3p.** (**A** and **B**) Subcellular distribution of circUBXN7 was determined by nuclear mass separation assay. (**C** and **D**) FISH assay for investigating the subcellular localization of circUBXN7 in T24 and UM-UC-3 cells. U6 was used as a nuclear control. Scale bar, 50μm. (**E**) The secondary structure of circUBXN7 that possibly binds to miR-1247-3p was predicted by RegRNA 2.0. The yellow region indicates the predicted motif structure. (**F**) The potential binding sites between circUBXN7 and miR-1247-3p were predicted by RNAhybrid. The red part represents the mutated base. (**G**) miR-1247-3p reduced the luciferase activity of circUBXN7 in 293T cells detected by dual-luciferase activity assay. (**H**) Anti-AGO2 RIP assay pulled down more circUBXN7 than in the anti-IgG group. (**I**) Relative miR-1247-3p levels immunoprecipitated by AGO2 in circUBXN7 overexpressing or control cells. (**J**) Relative miR-1247-3p levels immunoprecipitated by AGO2 or IgG in circUBXN7 overexpressing cells. (**K**) Colocalization of circUBXN7 and miR-1247-3p was detected in T24 cells by FISH assay. Scale bar, 50μm. *P<0.05, **P<0.01.

CircRNAs have been reported to mainly serve as competitive endogenous RNA (ceRNAs) [8], while several circRNAs have been verified to bind to or encode proteins [17–21]. Therefore, to elucidate the underlying action mechanism of circUBXN7 in BC, we first evaluated whether circUBXN7 contained an internal ribosome entry site (IRES) and an open reading frame (ORF), which are essential for encoding protein. By searching circRNADb (http://reprod.njmu.edu.cn/circrnadb), we found that hsa_circ_0001380 (CircBase ID) was in accordance with hsa_circ_24576 (circRNADb ID). The IRES elements were found at positions 42-191 and 107-247, but no ORF was found, indicating that the possibility of encoding protein was relatively low. Next, we evaluated its subcellular location. Nuclear mass separation assay and fluorescence *in situ* hybridization (FISH) verified that circUBXN7 predominantly resided in the cytoplasm of T24 and UM-UC-3 (Figure 4A-4D), implying that circUBXN7 may be involved in posttranscriptional regulation.

According to MiRanda, RegRNA 2.0 and RNAhybrid 2.2 prediction, circUBXN7 harbored a complementary sequence to miR-1247-3p (Figure 4E and 4F). To validate the interaction between circUBXN7 and miR-1247-3p, we constructed wild-type circUBXN7 sequences containing the predicted miR-1247-3p recognition sites or mutant sequences, which were then inserted downstream of the luciferase reporter gene in a psiCHECK-2 vector. After cotransfection of a luc-circUBXN7-wt plasmid with miR-1247-3p mimics or NC in 293T cells, dual-luciferase reporter assay was further performed. The results revealed that miR-1247-3p significantly reduced the luciferase activity of luc-circUBXN7-wt. However, no obvious change in luciferase activity was observed when cells were cotransfected with miR-1247-mimics and luc-circUBXN7-mut plasmid (Figure 4G), indicating that miR-1247-3p could interact with circUBXN7 through the complementary binding sites. In addition, according to previous studies, there is a prevalent phenomenon that miRNAs generally repress translation or degrade mRNA by binding with Argonaute 2 (AGO2) protein [15], so that both circRNAs and mRNAs could bind with AGO2 simultaneously [16,22,23]. To confirm the hypothesis that miR-1247-3p could directly bind to circUBXN7, we performed anti-AGO2 immuno-precipitation (RIP) assay to detect circUBXN7 levels in the immunoprecipitates pulled-down by anti-AGO2 antibody or nonspecific anti-IgG antibody in 293T cells. Indeed, endogenous circUBXN7 was efficiently enriched in cells carrying exogenous miR-1247-3p compared with anti-IgG group (Figure 4H). MiR-1247-3p pulled-down by AGO2 in circUBXN7-overexpressing cells was about 4-fold more than that in empty vector-transfected cells (Figure 4I). In addition, higher level of miR-1247-3p was observed when captured by AGO2 than IgG in circUBXN7-overexpressing cells (Figure 4J). Furthermore, FISH assay verified that circUBXN7 and miR-1247-3p were colocalized in T24 cells (Figure 4K). Collectively, these data indicated that circUBXN7 directly binds to miR-1247-3p and might function as miR-1247-3p sponge.

### CircUBXN7 antagonizes miR-1247-3p-mediated enhancement of cell proliferation, migration and invasion in BC cells

**Figure 5:**
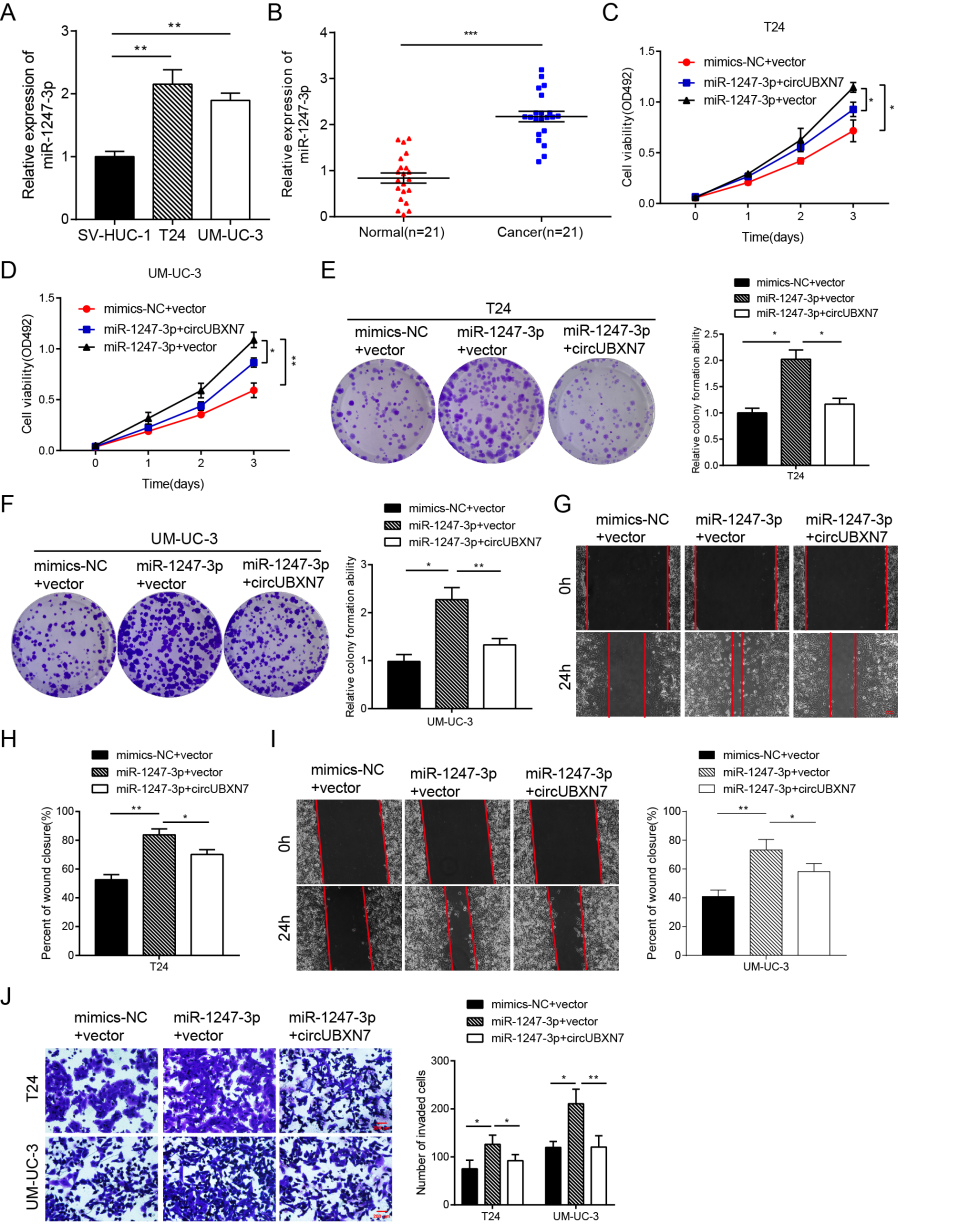
**CircUBXN7 reversed the miR-1247-3p-induced increase in proliferation, migration and invasion in BC cells.** (**A**) Relative expression of miR-1247-3p in BC cell lines was examined by qRT-PCR. (**B**) qRT-PCR analysis of miR-1247-3p expression in BC and normal tissues. (**C** and **D**) MTS assay indicated that miR-1247-3p elevated cell viability of BC cells and the effect was abolished by circUBXN7. (**E** and **F**) Colony formation assay showed that miR-1247-3p increased clone numbers of BC cells and the effect was relieved by circUBXN7. (**G**-**I**) Wound healing assay revealed that miR-1247-3p enhanced the migratory ability of BC cells and this effect was abrogated by circUBXN7. Scale bar, 500 μm. (**J**) Transwell Matrigel invasion assay demonstrated that miR-1247-3p augmented the invasive capacity of BC cells and this effect was eliminated by circUBXN7. Scale bar, 200 μm. *P<0.05, **P<0.01, ***P<0.001.

MiR-1247-5p (previous ID: hsa_miR-1247) has been reported to act as an onco-miR in castration-resistant prostate cancer [24] and as a tumor suppressor in non-small-cell lung cancer [25] and hepatocellular carcinoma [26]. However, studies have seldom been focused on miR-1247-3p. A recent study showed that tumor-derived exosomal miR-1247-3p could promote cancer progression and foster lung metastasis of liver cancer [27], but the role of miR-1247-3p in BC is still unknown. To investigate the role of miR-1247-3p in BC, qRT-PCR was first used to detect miR-1247-3p expression in BC cell lines and tissues. The expression of miR-1247-3p was significantly higher in T24 and UM-UC-3 than in SV-HUC-1 cells (Figure 5A). Based on PCR results of 21 paired BC tissues and adjacent normal tissues, miR-1247-3p expression was differentially upregulated in BC tissues in comparison with matched adjacent normal tissues (Figure 5B). The levels of miR-1247-3p were then increased by transfecting miR-1247-3p mimics into both T24 and UM-UC-3 cells. The MTS assay showed that higher levels of miR-1247-3p enhanced cell viability after transfection with miR-1247-3p mimics in T24 and UM-UC-3 cells (Figure 5C and 5D). Analogously, higher miR-1247-3p levels strikingly increased the colony numbers of BC cells (Figure 5E and 5F). However, the proliferation-promoting effects induced by exogenous miR-1247-3p were partially abolished by overexpression of circUBXN7 (Figure 5C-5F). Moreover, wound healing and Matrigel invasion assays confirmed that the migratory and invasive capacities were apparently increased in the miR-1247-3p mimics-transfected group compared with those in the NC group (Figure 5G-5J). Similarly, cell migratory and invasive capabilities were neutralized when cells were cotransfected with miR-1247-3p mimics and circUBXN7 compared with cotransfection with miR-1247-3p mimics and empty vector (Figure 5G-5J). In contrast, transfection with miR-1247-3p inhibitor in BC cells showed that the knockdown of miR-1247-3p strikingly reduced cell proliferation, migration and invasion, and such tumor suppressive effects were reversed by si-circUBXN7 (data not shown). Collectively, these results clearly demonstrated that miR-1247-3p acts as an onco-miR in BC, and circUBXN7 exerts its anti-tumor effects by sponging miR-1247-3p.

### CircUBXN7 suppresses proliferation and invasion through modulation of the miR-1247-3p/B4GALT3 axis in BC cells

**Figure 6:**
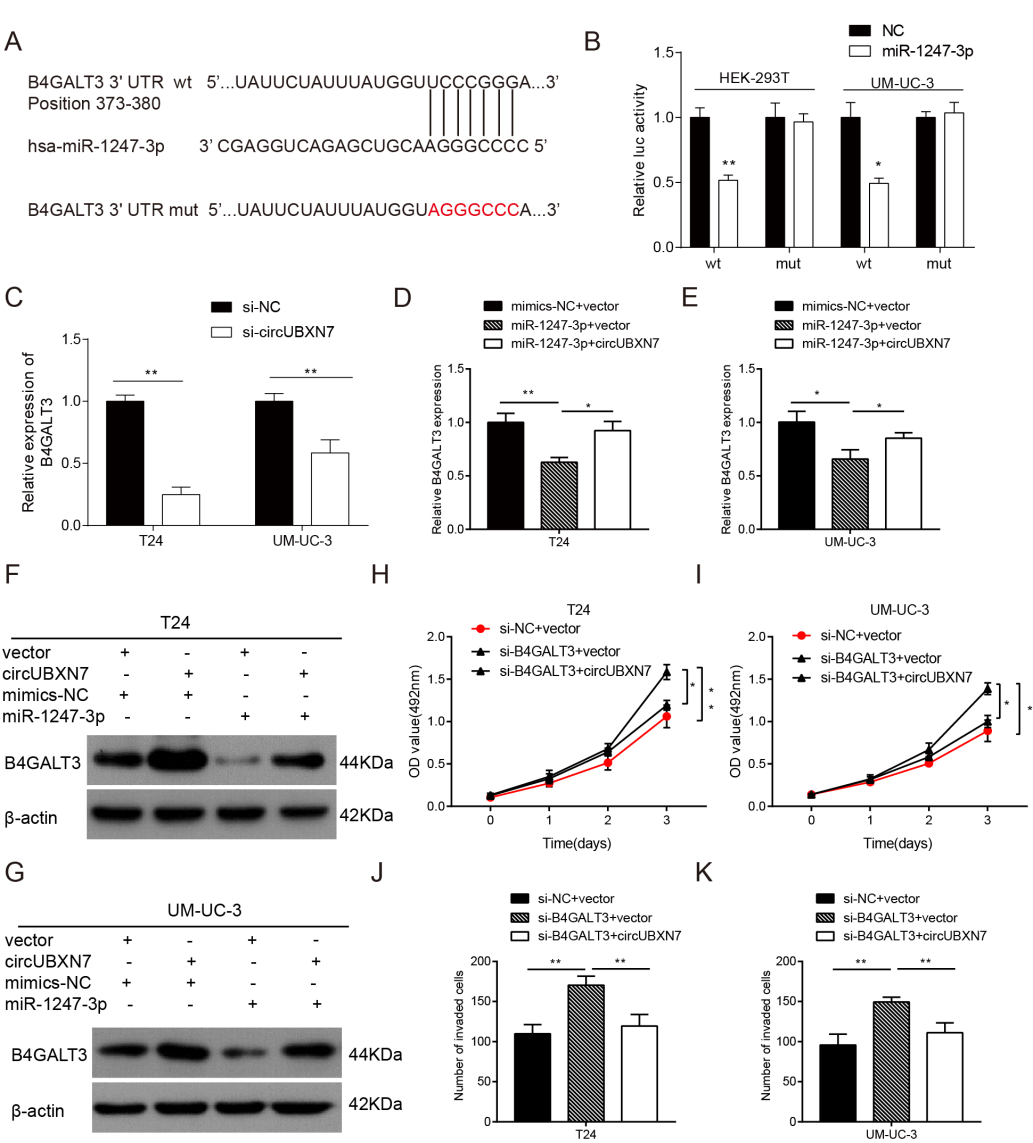
**CircUBXN7 antagonized miR-1247-3p-mediated repression of B4GALT3 expression.** (**A**) The potential binding sites between miR-1247-3p and B4GALT3 was predicted by Targetscan. The red part indicates the mutated base. (**B**) Dual-luciferase reporter assay showed that B4GALT3 was directly targeted by miR-1247-3p. (**C**) The effect of si-circUBXN7 on the mRNA level of B4GALT3 was determined by qRT-PCR. (**D** and **E**) CircUBXN7 reversed the suppressive effect of miR-1247-3p on B4GALT3 expression at the mRNA level. (**F** and **G**) CircUBXN7 neutralized miR-1247-3p-induced inhibition on B4GALT3 expression at the protein level. (**H** and **I**) MTS assay showed that the proliferation-promoting effect of si-B4GALT3 was reversed by circUBXN7. (**J** and **K**) Transwell Matrigel invasion assay indicated that circUBXN7 counteracted the invasive ability of BC cells transfected with si-B4GALT3. *P<0.05, **P<0.01.

To further study the downstream genes regulated by circUBXN7, Targetscan was first utilized to predict the potential targets of miR-1247-3p. As depicted in Figure 6A, the 3’UTR of βeta-1, 4-galactosyltransferase 3 (B4GALT3) was predicted to bind to miR-1247-3p with a high score. To confirm the prediction, the B4GALT3 3’UTR and its mutated sequence were separately cloned downstream of luciferase gene in the reporter vector psiCHECK-2 (Figure 6A). Then, a dual-luciferase reporter assay was performed. The results showed that overexpression of miR-1247-3p evidently decreased luciferase activity of the wild-type B4GALT3 in both 293T and UM-UC-3 cells, whereas almost no significant change in luciferase activity was detected in the mutant B4GALT3 group (Figure 6B). The results confirmed that B4GALT3 was directly targeted by miR-1247-3p. However, it was still unknown whether circUBXN7 could regulate miR-1247-3p-mediated repression of B4GALT3. Therefore, we silenced circUBXN7 expression in T24 and UM-UC-3 cells and examined the B4GALT3 mRNA expression by qRT-PCR. As expected, the B4GALT3 mRNA level was strikingly reduced in the circUBXN7-silenced group compared to that in NC group (Figure 6C). On the other hand, circUBXN7 overexpression significantly reversed miR-1247-3p-mediated suppression of B4GALT3 mRNA expression in both T24 and UM-UC-3 cells (Figure 6D and 6E). Western blotting revealed that circUBXN7 promoted B4GALT3 expression at the protein level, while miR-1247-3p dramatically reduced B4GALT3 expression, and this effect was partially counteracted by circUBXN7(Figure 6F and 6G). Next, we studied whether circUBXN7 could affect cell function through B4GALT3. MTS assay clearly showed that knockdown of B4GALT3 elevated cell viability of T24 and UM-UC-3 cells, which was reversed by circUBXN7 (Figure 6H and 6I). Analogously, circUBXN7 also diminished the invasive ability of cells induced by si-B4GALT3 (Figure 6J and 6K). Collectively, this evidence revealed that circUBXN7 could positively regulate B4GALT3 expression through sponging miR-1247-3p.

### Overexpression of circUBXN7 represses tumor growth *in vivo*

**Figure 7:**
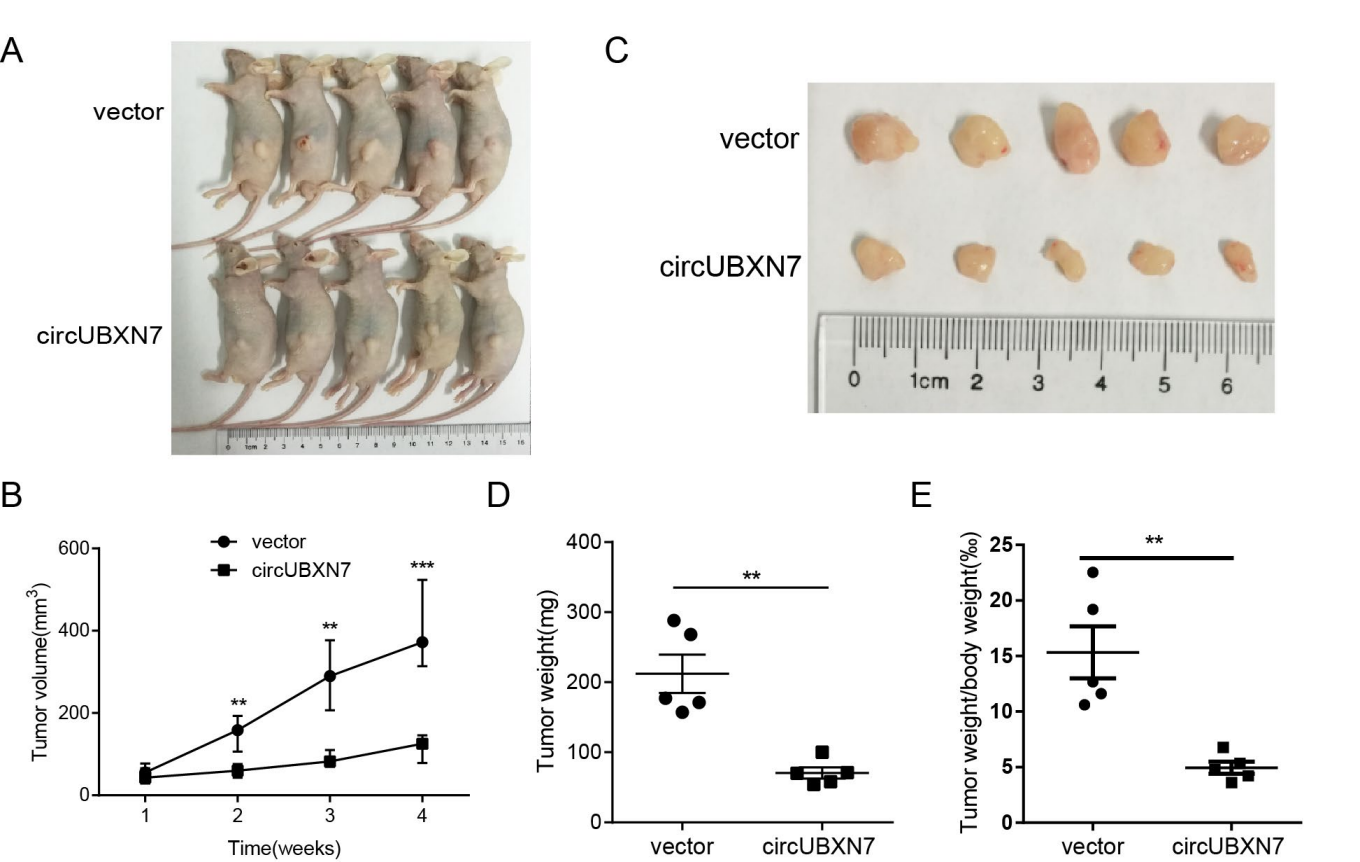
**Overexpression of circUBXN7 repressed tumor growth *in vivo*.** (**A**) The image of BALB/c nude mice inoculated with UM-UC-3 cells transfected with circUBXN7 or a control vector. (**B**) Subcutaneous tumor volume was calculated every week. (**C**) Image of the resected xenograft tumors. (**D**) Tumor weight was evaluated in circUBXN7- or control vector-treated mice. (**E**) The ratio of tumor weight/body weight was calculated in circUBXN7- or control vector-treated mice. **P<0.01, ***P<0.001.

To explore the biological function of circUBXN7 *in vivo*, we developed a xenograft tumor model and investigated the effect of circUBXN7 on BC tumor growth. Stably overexpressed circUBXN7 UM-UC-3 cells or control cells were subcutaneously injected into each flank of BALB/c nude mice. After inoculation for 30 days, the subcutaneous tumors were clearly visible (Figure 7A) and were harvested for tumor weight evaluation. Overexpression of circUBXN7 significantly suppressed xenograft tumor growth. A decrease in tumor volume, tumor weight and the ratio of tumor weight and body weight were found in the circUBXN7 overexpression group compared with the control group (Figure 7B-7E).

## DISCUSSION

In recent years, circRNAs have attracted extensive attention due to their participation in numerous cellular physiobiological processes and critical roles in regulating gene expression [8,16,]. Based on high-throughput RNA sequencing, many dysregulated circRNAs have been identified in BC tissues compared to nontumor tissues [16]. However, the roles and underlying mechanisms of action of many circRNAs in BC remain elusive. In the present study, we identified that a novel circular RNA circUBXN7 acted as miR-1247-3p sponge to suppress BC growth. Our study revealed a vital role of the circUBXN7-miR-1247-3p-B4GALT3 regulatory network in BC progression. To the best of our knowledge, this is the first report to generally investigate the expression, biological function and action mechanism of circUBXN7 in BC.

The majority of circRNAs are directly back-spliced from the exons of precursor mRNA [16]. In the current study, we identified one of the transcripts derived from exons 2, 3 and 4 of the UBXN7 gene (termed circUBXN7). RNase R treatment and Actinomycin D assay verified the high stability of circUBXN7 in BC cells. CircRNAs are not simply the by-products of mis-splicing or splicing errors [8,16], implying that circRNAs might play an important role in BC development. To date, accumulating evidence has shown that several circRNAs are involved in the tumorigenesis and progression of BC. For instance, circular RNA MYLK promotes BC progression by activating the VEGFA/VEGFR2 and Ras/ERK signaling pathways [15]. Similarly, circ-BPTF promotes BC growth by sponging miR-31-5p [28]. These circRNAs are exemplified as oncogenes contributing to BC progression. However, circRNAs also exert tumor suppressive roles in BC. For example, circHIPK3 sponges miR-558 to suppress migration, invasion and angiogenesis of BC through downregulation of heparanase expression [13]. Circ-ITCH inhibits BC progression by sponging miR-17/miR-224 and regulating p21 and PTEN expression [14]. In our study, we also found that circUBXN7 was distinctly downregulated in not only BC tissues but also BC cell lines. It has been reported that RNA-binding proteins could broadly regulate the biogenesis of circRNAs [29]. DHX9, an abundant nuclear RNA helicase, contributes to the downregulation of circSMARCA5 in hepatocellular carcinoma by binding to inverted-repeat Alu elements [30,31]. Whether downregulation of circUBXN7 in BC is also regulated by RNA-binding proteins should be further investigated in our future study.

CircRNAs are derived from exonic, intronic or intergenic regions [7,16]. The circRNAs spliced from introns or exon-introns are primarily located in the cell nucleus, and play essential roles via specific RNA-RNA interactions [32,33]. However, exon-derived circRNAs generally accumulate in cytoplasm [16]. Cytoplasmic noncoding RNAs may be involved in posttranscriptional effects on mRNA stabilization or translation [8,16]. Consistently, circUBXN7 arose from three exons of the UBXN7 gene, and the subcellular fraction isolation assay and FISH analysis revealed that circUBXN7 was mostly located in the cytoplasm, implying that it may function posttranscriptionally. It has been highlighted recently that circRNAs harbor many potential miRNA response elements and function as competing endogenous RNAs for miRNAs in various cancers. For example, the well-known ciRS-7 contains more than 70 binding sites of miR-7 and functions as a sponge of miR-7 [8]. In the present study, we proved that circUBXN7 directly interacted with miR-1247-3p through the complementary binding sites. Moreover, rescue experiments validated that overexpression of circUBXN7 could partially abrogate the oncogenic effects induced by miR-1247-3p in BC. These results indicate that circUBXN7 plays a tumor suppressive role in the malignant biological behavior of BC cells by serving as a competitive endogenous RNA (ceRNA) of miR-1247-3p.

In our further study, we investigated whether circUBXN7 could regulate B4GALT3, the target gene of miR-1247-3p. B4GALT3 has been reported to play different roles in different diseases including human cancers. For example, B4GALT3 was significantly overexpressed in dup (1q)-positive acute lymphoblastic leukemias (ALLs) compared with high hyperdiploid ALLs without dup (1q) [34]. In neuroblastoma, overexpression of B4GALT3 increased migration, invasion, and tumor growth of neuroblastoma cells, and B4GALT3 expression was correlated with advanced clinical stages, unfavorable Shimada histology, and lower survival rate [35]. Moreover, B4GALT3 enhanced β1-integrin stability and contributed to the oncogenic activity in cervical cancer cells [36]. However, the role of B4GALT3 in different human tissues is controversial. For instance, B4GALT3 suppressed cell invasion, and enhanced cell adhesion to laminin in HTR8/SVneo cells [37]. Besides, B4GALT3 overexpression suppressed cell migration, invasion and adhesion in colorectal cancer (CRC), and B4GALT3 was negatively correlated with poorly differentiated histology, advanced stages, regional lymph node metastasis and distant metastasis in CRC patients [38]. In addition, the knockdown of B4GALT3 promoted fibroblast motility and led to an activation of β1-integrin-NF-κB signaling in fibroblasts, further promoting liver cancer progression by secreting interleukin (IL)-6 and IL-8 [27]. These findings demonstrated that B4GALT3 may function as an oncogene or tumor suppressor in different kinds of cancers. However, the role of B4GALT4 has not been studied in BC before. The controversial roles of B4GALT3 in human cancers compelled us to elucidate its biological function in BC. In our study, we confirmed that silencing of B4GALT3 significantly enhanced cell viability and invasive capacity, indicating that B4GALT4 exerted a tumor suppressive role in BC. However, how B4GALT3 expression was modulated during BC progression remained unclear. Our results further verified that B4GALT3 was a direct target of miR-1247-3p, which was in line with the results of Fang’s study [27]. MiR-1247-3p was confirmed to play oncogenic roles in BC and showed inhibitory effects on B4GALT3 expression. These effects could be partially abolished by circUBXN7 overexpression, which demonstrated that circUBXN7 was involved in ceRNA regulatory network and served as miR-1247-3p sponge to modulate B4GALT3 expression.

In conclusion, for the first time, we provided direct clinical and biomedical evidence that decreased circUBXN7 was associated with a poor prognosis in BC patients. We further demonstrated that circUBXN7 was a tumor suppressor in BC by sponging miR1247-3p and upregulating B4GALT3 expression. Therefore, our findings provide a better understanding of circUBXN7 function in BC progression and revealed that circUBXN7 may serve as potential target for BC treatments in the future.

## MATERIALS AND METHODS

### Patients and clinical specimens

A cohort of 84 cases of BC tissues including 30 paired BC tissues and adjacent nontumor tissues were collected from patients who underwent surgery and were diagnosed with urothelial carcinoma of bladder at Sun Yat-sen Memorial Hospital from August 2010 to October 2016. All tissue samples were frozen in liquid nitrogen immediately after dissection from patients. This study was approved by the ethics committee of Sun Yat-sen Memorial Hospital, Sun Yat-sen University. Written informed consent was obtained from all patients.

### Cell culture

All cell lines including 293T, human immortalized uroepithelium cell SV-HUC-1 and human BC T24 and UM-UC-3 were purchased from ATCC. All media were mixed with 10% fetal bovine serum (BI, Israel) and 1% penicillin/streptomycin (Gibco, USA). The 293T and UM-UC-3 cells were maintained in DMEM medium (Gibco, USA), T24 cells were cultured in RPMI 1640 medium (Gibco, USA) and SV-HUC-1 were cultured in F-12K medium (Gibco, USA). Then, the cells were kept in a water-saturated incubator under an atmosphere of 5% CO_2_ at 37°C.

### siRNA, plasmid construction and cell transfection

Transfection of miRNA mimics, miRNA inhibitor and siRNAs (GenePharma, China, http://www.genepharma.cn/) were performed using Lipofectamine RNAimax (Invitrogen, USA). To construct a circUBXN7 plasmid, full-length human circUBXN7 cDNA was synthesized and cloned into plenti-ciR-GFP-T2A vector (IGE Biotech Co, China, http://www.igebio.com/zh-hans). For luc-circUBXN7 plasmid, the linear form of circUBXN7 sequence was synthesized and cloned downstream of luciferase gene in psiCHECK-2 (Synbio, China, http://www.synbio-tech.com.cn/). All plasmids were transfected into cells using X-treme (Sigma, USA) according to the manufacturer’s instructions. All sequences used in this study are listed in the supplementary file (Table S1).

### RNA extraction, gDNA extraction and quantitative real-time PCR (qRT-PCR)

RNA was extracted using RNAiso (Takara, Japan), and gDNA was extracted from cells by Universal Genomic DNA Kit (CWBIO, China, http://www.cwbiotech.com/) according to the manufacturer’s recommendations. For circRNA and mRNA detection, cDNA was reverse transcribed using Prime Script RT Master Mix (Takara, Japan). For miRNA examination, cDNA was synthesized by miRNA First Strand cDNA Synthesis kit (Sangon Biotech, China, http://www.sangon.com/). Quantitative real-time PCR was performed on Quantstudio™ DX system (Applied Biosystems, Singapore). The expression of circRNA or mRNA was normalized to GAPDH, and small nuclear U6B (RNU6B) was used to normalize miRNA. The relative expression levels were calculated according to 2^-∆∆CT^ method. All primer sequences used in this study are listed in the supplementary file (Table S2).

### RNase R treatment and Actinomycin D assay

RNase R (Epicentre Technologies, USA) treatment was performed to degrade linear RNA. Briefly, 2 μg of total RNA was incubated for 30 min at 37°C using RNase R (3 U/μg). For Actinomycin D assay, 1×10^5^ T24 cells were seeded into 6-well plate and incubated for 24 h. Then, 2 mg/L Actinomycin D (Sigma, USA) was added into each well for 4, 8, 12 and 24 h respectively. Afterwards, the treated cells were harvested at the indicated time points for qRT-PCR analysis.

### Nuclear mass separation assay

RNA isolation of nuclear and cytoplasmic fractions were performed using NE-PER Nuclear and Cytoplasmic Extraction Reagents (Thermo Scientific, USA). Briefly, 1×10^7^ T24 and UM-UC-3 cells were harvested and suspended with 500 μL ice-cold Cell Fraction Buffer. After incubating on ice for 10 min, the suspension was centrifuged at 4 °C and 500×g for 5 min to separate the nuclear and cytoplasmic cell fractions. Then, the supernatant containing the cytoplasmic fraction was carefully aspirated away from the nuclear pellet, meanwhile, the nuclear pellet was lysed with 500 μL ice-cold Cell Disruption Buffer. Afterwards, the suspension of cytoplasmic fraction and nuclear lysate were immediately mixed with an equal volume of 2×Lysis/Binding Solution respectively for RNA isolation according to the manufacturer’s instructions.

### MTS assay

The transfected cells were seeded into 96-well plates at a density of 5×10^3^ cells per well and cultured for 1, 2, 3, and 4 days. Twenty microliter of MTS solution (Promega, USA) was added to each well at the indicated time. After incubation at 37°C for 2 h, optical density at 492 nm was measured using SPARK 10M spectrophotometer (Tecan, Austria).

### Colony formation assay

The treated cells were replated in 6-well plates at the density of 5×10^2^ per well and maintained for 10 days. Then, cells were fixed with 4% paraformaldehyde and stained with 0.1% crystal violet for 30 min.

### Wound healing assay

Transfected cells were cultured in 6-well plates and scraped using 200 μL pipette tips. At least three artificial wounds were photographed using 10 high-power fields at 0 and 24 h after scratch.

### Transwell Matrigel invasion assay

Transwell invasion assay was performed using a 24-well transwell chamber with 8-μm pore size (Corning, USA). Transfected cells were suspended with 200 μL of serum-free medium and plated onto the precoated Matrigel (#356234, BD Bioscience) in the upper chamber, and 600 μL of medium containing 10% FBS was added to the lower chamber. After incubation at 37°C with 5% CO_2_ for 11 h (for T24) and 24 h (for UM-UC-3), cells that invaded to the lower membrane surface of the filter were fixed with 4% paraformaldehyde and stained with 1% crystal violet. The number of invaded cells were calculated in at least three random fields.

### Fluorescence *in situ* hybridization (FISH)

FISH assay was conducted according to the previously reported methods [16]. Briefly, after hybridization with Cy3-labeled circUBXN7 probe and Cy5-labeled miR-1247-3p probe (GenePharma, China) at 37°C overnight, Hoechest 33342 was used to counterstain the nuclei. Then, the images were captured on ZEISS LSM800 Confocal Microscope (Carl Zeiss AG, Germany).

### Luciferase activity assay

Cells were seeded into 24-well plates at a density of 5×10^4^ cells per well the day before transfection. Cells were transiently cotransfected with psiCHECK-2-circUBXN7 (Synbio, China, http://www.synbio-tech.com.cn/) and miRNA mimics or control mimics for 48 h, the Renilla luciferase activity was measured using dual-luciferase reporter assay system (Promega, USA) following the manufacturer′s instructions.

### RNA immunoprecipitation

RIP assay was performed using Magna RIP RNA-Binding Protein Immunoprecipitation Kit (Millipore, USA) according to the manufacturer′s protocols. Anti-AGO2 antibody (Cell Signaling Technology, USA) and normal IgG (Millipore, USA) were used for immunoprecipitation. The purified RNA was then subjected to qRT-PCR.

### Animal studies

All animal experiments were performed with the approval of the Animal Ethics Committee of Sun Yat-sen University. Four-week-old female BALB/c nude mice were purchased from the Experimental Animal Center of Sun Yat-sen University. For the *in vivo* tumor growth study, a total of 5×10^6^ UM-UC-3 cells stably transfected with Lenti-circUBXN7 or Lenti-empty vector was subcutaneously injected into the left flank abdomen of nude mice (n=5 for each group). The tumor volumes were calculated by (length× width^2^)/2 every week after injection. Four weeks later, all mice were sacrificed. All subcutaneous tumors were resected for tumor weight measurement and tumor size evaluation.

### Western blotting

Western blotting was conducted using an SDS-PAGE electrophoresis system according to published methods [6].The primary antibodies specific for B4GALT3 (1:1000 dilution, Proteintech, USA), β-actin(1:10000 dilution, Proteintech, USA) were incubated at 4°C overnight. Thereafter, signals were detected using Immobilon ECL substrate (Millipore, Germany), and the images were obtained by Optimax X-ray Film Processor (Protec, Germany).

### Statistical analysis

All data were analyzed using SPSS 17.0 software (SPSS Inc., Chicago, IL, USA) or GraphPad prism 7 software (GraphPad Software, Inc., LaJolla, CA, USA). All data were presented as the mean±standard deviation. The chi-square test was applied to analyze the relationship between circUBXN7 expression and clinicopathological parameters. Two-tailed Student’s t-test was implemented to appraise the difference between two independent groups. One-way ANOVA analysis was used for comparisons of multiple groups. The Kaplan-Meier method was applied to plot the overall survival curves, and the log-rank test was used for evaluating the differences between groups. *P*<0.05 was set for statistical significance.

## SUPPLEMENTARY MATERIAL

Supplementary Table S1

Supplementary Table S2
