# Supplementary material for: Circular RNA circUBXN7 represses cell growth and invasion by sponging miR-1247-3p to enhance B4GALT3 expression in bladder cancer
Source: Aging (Albany NY). 2018 Oct 12;10(10):2606–23. doi: 10.18632/aging.101573 (PMC6224258; doi:10.18632/aging.101573)
Supplement: Supplementary Table S1 [file aging-10-101573-s001.docx]

**Table S1. The sequences of siRNAs used in this study.**

| **Name Sequences 5’-3’** | |
| --- | --- |
| si-NC sense | UUCUCCGAACGUGUCACGUTT |
| si-NC antisense | ACGUGACACGUUCGGAGAATT |
| si-circUBXN7 sense | GCAGCUUUGAAACAAGAAGTT |
| si-circUBXN7 antisense | CUUCUUGUUUCAAAGCUGCTT |
| si-UBXN7 sense | CAGAAAGTCTCCCCACAAAGATT |
| si-UBXN7 antisense | TCTTTGTGGGGAGACTTTCTGTT |
| si-B4GALT3 sense | GCGCCAGCAGCUUGCUUAUTT |
| si-B4GALT3 antisense | AUAAGCAAGCUGCUGGCGCTT |
