# Supplementary material for: Circular RNA circUBXN7 represses cell growth and invasion by sponging miR-1247-3p to enhance B4GALT3 expression in bladder cancer
Source: Aging (Albany NY). 2018 Oct 12;10(10):2606–23. doi: 10.18632/aging.101573 (PMC6224258; doi:10.18632/aging.101573)
Supplement: Supplementary Table S2 [file aging-10-101573-s002.docx]

**Table S2. The sequences of primers used in this study.**

| **Name Primers 5’-3’** | |
| --- | --- |
| circUBXN7-F | CCACCCATTGATTTGATGC |
| circUBXN7-R | CCGTCGTCTTTTAGGAGCAC |
| linear UBXN7-F  linear UBXN7-R | GGATAGCCGCTCAGATGAAG  TTTGTGGGGAGACTTTCTGG |
| GAPDH-F | GGAGCGAGATCCCTCCAAAAT |
| GAPDH-R | GGCTGTTGTCATACTTCTCATGG |
| hsa-miR-1247-3p | GGAACGUCGAGACUGGAGC |
| U6-F | CTCGCTTCGGCAGCACA |
| U6-R | AACGCTTCACGAATTTGCGT |
| miR reverse | Sangon Biotech, China |
